# Intravitreal ranibizumab as an adjunct to conventional surgery for neovascular glaucoma: a retrospective observational study of efficacy and clinical characteristics of surgical failure cases

**DOI:** 10.3389/fphys.2026.1915879

**Published:** 2026-09-15

**Authors:** Dan Li, Chao Li, Qing Ye

**Affiliations:** Department of Ophthalmology, Suzhou Kowloon Hospital Shanghai Jiao Tong University School of Medicine, Suzhou, Jiangsu, China

**Keywords:** conventional surgery, efficacy, intravitreal injection, neovascular glaucoma, ranibizumab

## Abstract

**Background:**

Intractable neovascular glaucoma (NVG), secondary to retinal ischemia, responds poorly to traditional surgery due to postoperative neovascularization and scarring. Intravitreal ranibizumab(IVR) swiftly alleviates neovascularization to enable subsequent treatment, yet its long-term efficacy remains unclear.

**Aim:**

This study sought to evaluate the ranibizumab-surgery combination therapy in NVG and describe clinical characteristics of surgical failure cases.

**Methods:**

This retrospective propensity score matching (PSM) cohort study enrolled 62 patients (62 eyes) with NVG between August 2022 and August 2024. Using 1:1 nearest-neighbor PSM, 31 eyes received preoperative IVR plus conventional surgery (study group), while 31 underwent surgery alone (control group). Over 12 months of follow-up, the primary endpoint was the 12-month surgical success rate; secondary outcomes included IOP, BCVA, postoperative NVI status, and complications. IOP and BCVA were analyzed using linear mixed-effects models, and clinical characteristics of the failure cases were described descriptively.

**Results:**

After PSM, baseline characteristics were generally comparable between groups, with residual imbalances noted for glaucoma stage and angle status. The study group showed significantly lower IOP at all postoperative visits (P < 0.05). The 12-month surgical success rate was numerically higher in the study group (93.5% vs. 77.4%). Secondary outcomes, including BCVA improvement and favorable NVI-free status, were more favorable in the study group, while the difference in overall complications did not reach statistical significance. Among the 9 failure cases, the majority had poor baseline BCVA, diabetes mellitus, and advanced glaucoma stage.

**Conclusion:**

In this retrospective matched cohort, adjunctive IVR was associated with more favorable IOP control and NVI-free status, whereas the difference in the 12-month surgical success rate, although numerically higher, did not reach statistical significance. Given the exploratory nature of this single-center study, larger prospective studies are warranted to confirm these findings.

**Significance and innovation:**

This retrospective matched cohort study suggests that perioperative ranibizumab may be associated with more favorable IOP control and NVI-free status, although the 12-month surgical success rate did not reach statistical significance. These findings are exploratory and require confirmation in larger prospective studies.

## 1 Introduction

Neovascular glaucoma (NVG) is a recalcitrant secondary glaucoma that frequently follows ischemic retinal diseases such as diabetic retinopathy and central retinal vein occlusion (Dumbrăveanu et al., 2021; Urbonavičiūtė et al., 2022; Aktas, 2025). Hallmarked by iris and angle neovascularization with subsequent aqueous outflow obstruction, NVG typically manifests as severe ocular pain and a precipitous rise in intraocular pressure (IOP). Without timely intervention, it can rapidly progress to optic atrophy, irreversible visual field defect, and eventually lead to blindness (Erdem et al., 2019; Iwasaki et al., 2022). In this study, “intractable NVG” is defined as eyes with intraocular pressure (IOP) > 24 mmHg despite the use of at least two classes of antiglaucoma medications, accompanied by active neovascularization of the iris and/or angle, and considered at high risk for surgical failure due to postoperative neovascularization and fibrosis. Due to the rapid progression of NVG and complex pathological changes, the clinical efficacy of conventional glaucoma surgery (such as trabeculectomy and glaucoma drainage valve implantation) is often limited, and postoperative complications such as NVI, filtering bleb scarring, and drainage tube obstruction are prone to occur, resulting in a high surgical failure rate (He et al., 2024; Pegu et al., 2025). According to literature reports, the 1-year success rate of conventional surgery alone for NVG is only 60%-80%, and some patients have poor vision improvement after surgery, which seriously affects the quality of life (He et al., 2017; Rajurkar et al., 2022).

The ophthalmic application of anti-vascular endothelial growth factor (VEGF) agents, intravitreal injection of ranibizumab has gradually become an important adjuvant treatment for NVG during the perioperative period (Wang et al., 2024). As a humanized anti-VEGF recombinant antibody fragment, ranibizumab is a widely used FDA-approved anti-VEGF agent for ocular diseases, and its overall effectiveness, safety and intravitreal injection mode of administration have been confirmed in a large number of studies (Zhao et al., 2023). Clinical practice has shown that preoperative injection of ranibizumab can quickly subside iris NVI, reduce ocular inflammatory response, reduce the risk of bleeding during surgery, and create more favorable ocular conditions for routine surgery (Lüke et al., 2013; Sun et al., 2017). However, the long-term efficacy of combining ranibizumab with conventional surgery for NVG remains a subject of debate. Some studies have a short follow-up time and lack long-term observation of visual prognosis and complications. At the same time, the key clinical factors affecting the prognosis of this combination therapy are not completely clear, which still need to be further verified by well-designed studies.

To address confounding, we employed propensity score matching (PSM) to balance baseline covariates between groups in 62 NVG patients. The study conducted a head-to-head comparison of 12-month outcomes between ranibizumab-augmented surgery and conventional surgery, with a descriptive analysis of clinical characteristics in failure cases. We explored whether the ranibizumab-surgery combination was associated with differences in IOP control, BCVA, and NVI regression compared with surgery alone, while evaluating the 12-month surgical success rate as the primary endpoint. Preoperative ranibizumab may be associated with more favorable NVI regression; recurrence and complication rates were also compared between groups. This study adds to the body of exploratory evidence on integrated NVG management.

## 2 Materials and methods

### 2.1 Study design

This investigation employed a retrospective propensity score matching (PSM) cohort design involving NVG patients from August 2022 to August 2024. A total of 94 patients were initially assessed for eligibility. Of these, 16 were excluded prior to PSM: 12 due to incomplete clinical data (missing baseline IOP records in 5 cases, missing BCVA records in 4 cases, and missing glaucoma stage in 3 cases), and 4 due to loss to follow-up within 12 months. The remaining 78 patients with complete data were available for PSM, comprising 40 patients in the study group and 38 in the control group. Using 1:1 nearest-neighbor matching without replacement (caliper = 0.2), 31 matched pairs (62 patients) were successfully identified; the remaining 16 patients (9 from the study group and 7 from the control group) were excluded because they did not match any counterpart within the specified caliper. The final matched cohort therefore comprised 31 patients in each group (**Figure 1**). All subjects completed the 12-month follow-up, as outlined in the study schematic (**Figure 1**).

**Figure 1:**
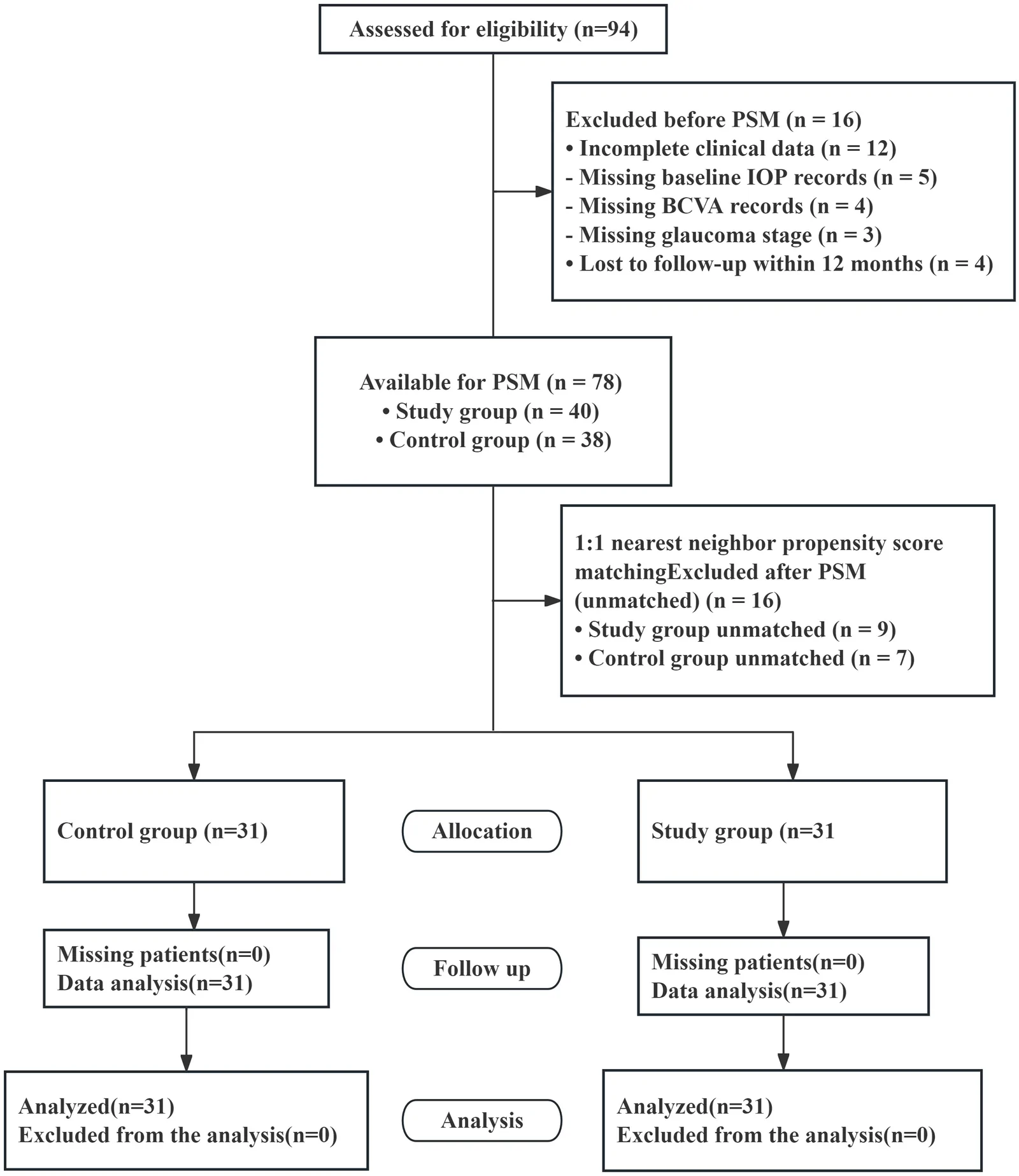
Design flow chart.

### 2.2 Ethical explanation

This study received ethics approval from our institutional committee, complying with the Declaration of Helsinki and international standards for medical research ethics. For this retrospective analysis, informed consent was exempted by the ethics board. Patient privacy was strictly maintained through full data anonymization.

### 2.3 Inclusion and exclusion criteria

Inclusion criteria: ① Fulfilled the established diagnostic criteria for NVG, unilateral onset (National Institute for Health and Care Excellence, 2022); ② Age of 18–80 years old, both sexes; ③ Presence of underlying retinal ischemia (e.g., diabetic retinopathy, retinal vein occlusion); ④ Preoperative BCVA < 0.1; ⑤ IOP > 24 mmHg (using at least two kinds of IOP lowering drugs); ⑥ Complete clinical data.

Exclusion criteria: ① Combined with secondary glaucoma caused by other causes (inflammatory glaucoma, traumatic glaucoma, lens-induced glaucoma). DR/CRVO-related retinal inflammation was not considered an exclusion criterion; ② Previous history of vitreoretinal surgery or anti-VEGF therapy within 6 months prior to the index surgery (date of the glaucoma procedure) (Kananen, 2025); ③ Severe corneal opacity or lens opacity affecting intraocular observation or surgical operation; ④ Accompanied by active ocular inflammation or infection; ⑤ Severe cardiovascular and cerebrovascular diseases, liver and kidney insufficiency or coagulation dysfunction; ⑥ Pregnant or lactating women.

Regarding panretinal photocoagulation (PRP), patients with prior PRP were not excluded, as PRP is a standard etiological treatment for retinal ischemia (the underlying cause of NVG). However, to minimize confounding, the proportion of patients with prior PRP was balanced between groups using propensity score matching. In the matched cohort, prior PRP was documented in 12 patients (38.7%) in the study group and 10 patients (32.3%) in the control group (P = 0.602). For patients without prior PRP or with incomplete PRP, additional PRP was performed during vitrectomy when indicated.

### 2.4 Treatment methods

The treatment plan of all patients was based on the actual clinical decision of the patient at the time of visit. Patients in the study group underwent conventional surgery combined with ranibizumab. The anti-VEGF agent (Lucentis^®^, 10 mg/ml, Novartis) was administered intravitreally one week preoperatively (Liu et al., 2016). This interval was selected based on previous studies demonstrating significant regression of iris neovascularization within 5–7 days after ranibizumab injection, which creates favorable conditions for subsequent surgery (Lüke et al., 2013; Gao et al., 2023). A one-week interval also allowed sufficient time for clinical reassessment of NVI regression and surgical planning. After that, the surgical methods were selected according to the specific conditions of the patients (Shchomak et al., 2019): compound trabeculectomy for eyes with clear media and open angle (or partially open angle after NVI regression); Ahmed glaucoma valve (AGV) implantation for extensive angle closure (>270°) or failed previous filtration surgery; and vitrectomy combined with PRP and trabeculectomy for eyes with dense vitreous hemorrhage, tractional retinal detachment, or media opacity precluding adequate PRP. These criteria were applied consistently to both groups. All surgical procedures were performed by the same surgical team following standardized institutional protocols. For compound trabeculectomy, mitomycin C (0.2 mg/mL for 3 minutes) was applied subconjunctivally in all cases to prevent bleb scarring. AGV implantation (model FP7) and vitrectomy (23-gauge or 25-gauge) were performed according to standard institutional protocols, with intraoperative modifications made as needed based on individual patient conditions. Specifically, in the study group, 17 eyes underwent compound trabeculectomy, 9 eyes AGV implantation, and 5 eyes vitrectomy combined with PRP and trabeculectomy. Control patients underwent conventional surgery alone, with identical surgical selection criteria as the study group, including compound trabeculectomy (20 eyes), Ahmed glaucoma valve implantation (7 eyes), vitrectomy combined with panretinal photocoagulation and trabeculectomy (4 eyes).

### 2.5 Observation indicators

By consulting the hospital electronic medical record system, image archiving and course and nursing records, the data of the following observation indicators were retrospectively extracted and collected.

#### 2.5.1 Baseline data

The patient’s age, gender, primary disease (proliferative diabetic retinopathy, central retinal vein occlusion, etc.), complications, IOP, preoperative BCVA, glaucoma stage, Angle neovascularization (NVA), NVI status, and surgical methods were extracted. All data were obtained from inpatient medical records and nursing records.

#### 2.5.2 Main outcome measures

1. IOP: the IOP values of the patients before operation and 1 week, 1 month, 3 months, 6 months, and 12 months after operation were extracted from the medical records. All the data were obtained from the measurement records of Goldmann applanation tonometer during outpatient or hospitalization.
2. The operative success rate was evaluated at the 12-month follow-up, adopting definitions aligned with recent NVG literature (Zhou et al., 2022; Ramji et al., 2023). Complete success was defined as IOP between 6 mmHg and 21 mmHg without antiglaucoma medications and without additional glaucoma surgery. Qualified success was defined as IOP between 6 mmHg and 21 mmHg with antiglaucoma medications (any number) and without additional glaucoma surgery. Failure was defined as IOP > 21 mmHg or < 6 mmHg despite maximal medical therapy, or the need for additional glaucoma surgery (e.g., cyclodestructive procedure, repeat drainage device implantation), or loss of light perception due to uncontrolled IOP.

#### 2.5.3 Secondary observation indicators

1. BCVA: Serial BCVA data were collected pre- and post-operation across all follow-up time points. The data were obtained from the standard logarithmic visual acuity chart examination and uniformly converted into LogMAR units for analysis (Kilani et al., 2025).
2. Postoperative NVI status was evaluated by reviewing slit-lamp microscopy records. NVI-free status was defined as the absence of NVI at the follow-up visit, including patients who had no NVI at baseline and remained NVI-free, as well as those with baseline NVI who achieved regression. For patients with NVI at baseline, regression was defined as complete disappearance of NVI or presence of only residual fibrotic vascular sheath. Recurrence was defined as reappearance of NVI that had subsided after surgery (Li et al., 2022). (NVA was not included in this endpoint due to inconsistent gonioscopic recording; see Limitations.)
3. Incidence of complications: Intraoperative and postoperative complications were extracted from course records, nursing records, and complication event reports, including hyphema, choroidal detachment, shallow anterior chamber, hypotony, drainage tube obstruction, and malignant glaucoma.

### 2.6 Sample size calculation

This was a retrospective analysis of a fixed clinical cohort. All patients who met the inclusion criteria during the study period were enrolled, and no formal prospective sample size calculation was performed. The final sample size of 31 patients per group after PSM was determined by the availability of eligible cases and successful matching, rather than by a prespecified power calculation. Recognizing the limited sample size, the study is considered exploratory in nature, and the findings should be interpreted as hypothesis-generating. The reported effect estimates and confidence intervals are provided to describe observed associations, which require confirmation in larger prospective studies.

### 2.7 Statistical analysis

Data extraction and analysis were performed using SPSS 26.0. To reduce selection bias, PSM was performed using a logistic regression model that included age, sex, primary etiology, baseline IOP, baseline BCVA, glaucoma stage, hypertension, and diabetes mellitus as covariates. Angle status was also included in the PSM model to ensure balance of this clinically important variable. Matching was conducted using a 1:1 nearest-neighbor algorithm without replacement, with a caliper width of 0.2 applied to the logit of the propensity score. The region of common support was assessed and confirmed. Balance between groups was evaluated using standardized mean differences (SMD). Consistent with recommended practice for matched cohorts, SMD < 0.3 was considered acceptable. Residual imbalances were noted for glaucoma stage and angle status; these variables were therefore additionally adjusted for in the longitudinal outcome models (see below). After PSM, all comparative analyses respected the matched-pair structure. The primary endpoint of this study was the 12-month surgical success rate. All other outcomes, including IOP at individual time points, BCVA, regression/recurrence of anterior-segment neovascularization (defined as postoperative NVI status; NVA was not included in the primary endpoint definition due to inconsistent gonioscopic recording, see Limitations), and complication rates, were considered secondary or exploratory.

For continuous outcomes (IOP and BCVA), linear mixed-effects models were applied with group, time point, and their interaction as fixed effects. To adjust for residual imbalances after PSM, the models included preoperative values (preoperative IOP for the IOP model; preoperative BCVA for the BCVA model), glaucoma stage, angle status, and age as covariates. To account for the matched-pair design and the hierarchical data structure—repeated measurements nested within patients, and patients nested within matched pairs—the model specified matched-pair ID as a random intercept and patient-nested-within-pair (MatchID × PatientID) as the repeated subject, with a first-order autoregressive [AR(1)] covariance structure for repeated measurements across time points within the same patient. The models were estimated using restricted maximum likelihood (REML) with Satterthwaite approximation for degrees of freedom. All models converged successfully; covariance-parameter estimates, including the AR(1) correlation coefficients, are reported in the model output (see **Supplementary Materials**). Estimated marginal means, adjusted between-group differences with 95% confidence intervals (CIs), and group-by-time interaction P values were reported. Pairwise comparisons at each time point were adjusted using the least significant difference (LSD) method; these secondary comparisons are exploratory and were not adjusted for multiplicity. Although the follow-up visits were not strictly equally spaced, the AR(1) structure was chosen as a parsimonious approach to model the declining correlation between repeated measures over time. To assess robustness, we performed a sensitivity analysis using an unstructured covariance matrix. For BCVA, the unstructured model converged and yielded results consistent with the primary analysis; for IOP, the unstructured model did not converge, likely due to the limited sample size. The AR(1) structure is supported by the strong within-patient temporal correlation (AR1 ρ = 0.972 for IOP; AR1 ρ = 0.958 for BCVA). The exact SPSS MIXED syntax is provided in the **Supplementary Materials** for full reproducibility.

For paired binary outcomes (surgical success, NVI-free status, and complications), McNemar’s test was used. To ensure reproducibility, we report the number of discordant pairs (B and C) for each endpoint, where B is the number of pairs in which the study group experienced the event while the control group did not, and C is the number of pairs with the reverse pattern. Risk differences were calculated as (B − C)/N, where N is the total number of matched pairs, and their 95% confidence intervals were estimated using the standard error of the paired difference, ensuring consistency with the McNemar test results. For the recurrence analysis, the denominator was restricted to patients with baseline NVI who achieved regression during follow-up and were therefore at risk of recurrence; patients who never achieved regression were not included in the recurrence denominator. Given the limited sample size, this analysis is reported descriptively rather than as a paired comparison.

For BCVA analysis, non-numeric visual acuities (counting fingers, hand motion, light perception, and no light perception) were converted to LogMAR values using the following standards: counting fingers = 2.0, hand motion = 2.3, light perception = 2.6, and no light perception = 2.9. Given the potential impact of this conversion, sensitivity analyses using the Wilcoxon signed-rank test were performed to confirm the robustness of the BCVA improvement findings. Due to the limited number of failure events (n = 9), formal multivariable prognostic analysis was not performed; instead, clinical characteristics of the failure cases were described descriptively. Continuous data are presented as mean ± standard deviation; categorical data as frequency (percentage). No adjustment for multiple comparisons was applied to secondary exploratory outcomes; therefore, these findings should be interpreted as hypothesis-generating rather than confirmatory. All tests were two-sided, and P < 0.05 was considered statistically significant.

## 3 Results

### 3.1 Comparison of baseline data

After propensity score matching, the two groups demonstrated generally acceptable balance, with most SMDs falling below the conventional threshold of 0.3 (**Table 1**). However, several covariates exhibited residual imbalances, including glaucoma stage (SMD = 0.265), angle status (SMD = 0.258), systolic blood pressure (SMD = 0.207), diastolic blood pressure (SMD = 0.214), age (SMD = 0.200), and baseline IOP (SMD = 0.199). The primary disease distribution was similar between groups, with proliferative diabetic retinopathy accounting for 51.6% in the study group and 58.1% in the control group, central retinal vein occlusion for 35.5% and 29.0%, respectively, and other ischemic retinopathies for 12.9% in both groups. The ‘other ischemic retinopathies’ category included ocular ischemic syndrome (2 cases in study group, 1 in control), branch retinal vein occlusion (1 case in control), and combined retinal artery-vein occlusion (1 case in control). Prior PRP was documented in 12 patients (38.7%) in the study group and 10 patients (32.3%) in the control group (SMD = 0.134). Preoperative antiglaucoma medication use (2.89 ± 0.66 vs. 2.79 ± 0.57) and systemic parameters, including HbA1c (7.94 ± 1.84% vs. 7.74 ± 1.88%), serum creatinine (73.16 ± 15.80 vs. 71.79 ± 16.27 μmol/L), were also comparable. To minimize potential confounding arising from these residual imbalances, glaucoma stage and angle status—the two variables with the largest imbalances—were additionally adjusted for as covariates in the longitudinal mixed-effects models for IOP and BCVA outcomes.

**Table 1:** Comparison of patient baseline characteristics after PSM.

Indicators	Study group (n=31)	Control group (n=31)	SMD	*P*-value
Demographics				
Age (years, mean ± SD)	57.3 ± 9.8	59.1 ± 8.5	0.196	0.442
Male, n (%)	17 (54.8%)	19 (61.3%)	0.131	0.607
Primary Disease, n (%)				0.935
Proliferative Diabetic Retinopathy	16 (51.6%)	18 (58.1%)	0.131	
Central Retinal Vein Occlusion	11 (35.5%)	9 (29.0%)	0.137	
Other Ischemic Retinopathies	4 (12.9%)	4 (12.9%)	0.000	
Comorbidities, n (%)				
Hypertension	15 (48.4%)	18 (58.1%)	0.194	0.445
Diabetes Mellitus	17 (54.8%)	19 (61.3%)	0.131	0.607
Ocular baseline characteristics				
Preoperative IOP (mmHg, mean ± SD)	51.0 ± 7.0	52.4 ± 7.2	0.199	0.474
Preoperative LogMAR BCVA (mean ± SD)	2.10 ± 0.42	2.11 ± 0.42	0.024	0.933
Glaucoma Stage, n (%)				0.297
Stage III	17 (54.8%)	21 (67.7%)	0.264	
Stage IV	14 (45.2%)	10 (32.3%)	0.264	
No. of Preop Antiglaucoma Meds (mean ± SD)	2.89 ± 0.66	2.79 ± 0.57	0.163	0.541
Angle Status, n (%)				0.309
NVA only	13 (41.9%)	17 (54.8%)	0.257	
NVA with NVI	18 (58.1%)	14 (45.2%)	0.257	
Surgical Procedure, n (%)				0.767
Combined Trabeculectomy	17 (54.8%)	20 (64.5%)	0.199	
Ahmed Glaucoma Valve Implant	9 (29.0%)	7 (22.6%)	0.147	
Combined Vitrectomy	5 (16.1%)	4 (12.9%)	0.091	
Prior treatment history, n (%)				
Prior PRP	12 (38.7%)	10 (32.3%)	0.135	0.596
Systemic control parameters				
HbA1c (%, mean ± SD)	7.9 ± 1.8	7.7 ± 1.9	0.107	0.687
Systolic BP (mmHg, mean ± SD)	136 ± 14	139 ± 15	0.207	0.414
Diastolic BP (mmHg, mean ± SD)	83 ± 9	85 ± 10	0.214	0.389
Serum creatinine (μmol/L, mean ± SD)	73.2 ± 15.6	71.8 ± 16.3	0.086	0.731

PSM, Propensity Score Matching; SD, Standard Deviation; IOP, Intraocular Pressure; BCVA, Best Corrected Visual Acuity; LogMAR, Logarithm of the Minimum Angle of Resolution; NVA, Neovascularization of the Angle; NVI, Neovascularization of the Iris; Preop, Preoperative; SMD, standardized mean difference (Cohen’s d for continuous variables; pooled proportion for categorical variables).

### 3.2 Changes in IOP

A linear mixed-effects model with group, time, and group-by-time interaction as fixed effects, adjusted for preoperative IOP, glaucoma stage, angle status, and age as covariates, revealed a significant main effect of group (F = 17.732, P < 0.001), a significant main effect of time (F = 71.708, P < 0.001), and a significant group-by-time interaction (F = 16.864, P < 0.001). Pairwise comparisons showed significantly lower IOP in the study group at all postoperative time points (all P < 0.01), with adjusted mean differences ranging from −2.76 mmHg (95% CI: −4.64 to −0.87) at 1 month to −5.27 mmHg (95% CI: −7.16 to −3.39) at 12 months (**Table 2**). Sensitivity analysis using an unstructured covariance structure to account for unequally spaced follow-up visits did not converge, likely due to the limited sample size; nevertheless, the AR(1) structure was supported by the strong within-patient temporal correlation (AR1 ρ = 0.972).

**Table 2:** Comparative analysis of IOP by group and timepoint (x ± s, mmHg).

Time	Study group (n=31)	Control group (n=31)	Adjusted mean difference (95% CI)	*P*-value
Preoperative	51.0 ± 7.0	52.4 ± 7.2	–	–
1 Week Postop	15.2 ± 2.6	18.9 ± 4.1	-3.76 (-5.65, -1.88)	<0.001
1 Month Postop	14.8 ± 2.9	17.5 ± 4.2	-2.76 (-4.64, -0.87)	0.005
3 Months Postop	15.5 ± 2.4	18.8 ± 3.4	-3.36 (-5.24, -1.48)	0.001
6 Months Postop	16.1 ± 2.6	20.3 ± 4.1	-4.26 (-6.14, -2.38)	<0.001
12 Months Postop	16.3 ± 2.5	21.5 ± 4.7	-5.27 (-7.16, -3.39)	<0.001

Data are presented as observed mean ± standard deviation. Estimated marginal mean differences (study group − control group) were derived from a linear mixed-effects model with group, time, and group-by-time interaction as fixed effects, adjusted for preoperative IOP, glaucoma stage, angle status, and age as covariates. To account for the matched-pair design and hierarchical data structure (repeated measurements nested within patients, and patients nested within matched pairs), the model included matched-pair ID as a random intercept and patient-nested-within-pair as the repeated subject with a first-order autoregressive [AR(1)] covariance structure for repeated measurements across time points within the same patient. The model included postoperative time points only (1 week, 1 month, 3 months, 6 months, and 12 months) as repeated measures; the preoperative row is shown for descriptive purposes only and was not included as an outcome in the model. Group main effect: F = 17.732, P < 0.001; Group × Time interaction: F = 16.864, P < 0.001. Pairwise comparisons were adjusted using LSD. IOP, intraocular pressure; CI, confidence interval.

### 3.3 Success rate of surgery

The study group achieved an overall success rate of 93.5% (29/31), compared with 77.4% (24/31) in the control group. In the paired analysis, the number of discordant pairs in which the study group succeeded while the control group failed was 7, and the number in which the study group failed while the control group succeeded was 2; the difference was not statistically significant (P = 0.180, McNemar’s test, **Table 3**). The complete success rate was 71.0% (22/31) in the study group versus 51.6% (16/31) in the control group (discordant pairs: B = 11, C = 5; P = 0.210). No significant difference was observed in qualified success rate between the two groups (discordant pairs: B = 5, C = 6; P = 1.000).

**Table 3:** Comparison of surgical success rates at 12 months postoperatively [n(%)].

Group	Study group (n=31)	Control group (n=31)	Number of inconsistent pairs (B/C) *	Risk difference (RD, %)	95% CI†	*P*-value‡
Complete Success	22 (71.0%)	16 (51.6%)	11/5	19.4%	−5.0%, 43.7%	0.210
Qualified Success	7 (22.5%)	8 (25.8%)	5/6	−3.2%	−24.1%, 17.7%	1.000
Overall Success	29 (93.5)	24 (77.4)	7/2	16.1%	−2.0%, 34.2%	0.180

*B = The number of pairs where the study group succeeded while the control group failed; C = The number of pairs where the study group failed but the control group succeeded. ^†^Calculated based on the paired difference using the Wald method to ensure consistency with the conclusion of the McNemar test. ^‡^McNemar test.

### 3.4 Changes in BCVA

A linear mixed-effects model with group, time, and group-by-time interaction as fixed effects, adjusted for preoperative BCVA, glaucoma stage, angle status, and age as covariates, revealed a significant main effect of group (F = 5.725, P = 0.024), a significant main effect of time (F = 21.771, P < 0.001), and a significant group-by-time interaction (F = 5.492, P < 0.001). Pairwise comparisons showed significantly better BCVA (lower LogMAR) in the study group from 3 months onward (P = 0.008 at 3 months, 0.007 at 6 months, and 0.005 at 12 months), with adjusted mean differences of −0.209 LogMAR (95% CI: −0.361 to −0.058) at 3 months, −0.213 LogMAR (95% CI: −0.364 to −0.062) at 6 months, and −0.226 LogMAR (95% CI: −0.377 to −0.074) at 12 months. The differences at 1 week and 1 month were not statistically significant (P = 0.326 and 0.071, respectively). The mean improvement from baseline to 12 months was significantly greater in the study group than in the control group (0.38 ± 0.53 vs. 0.16 ± 0.58 LogMAR; mean difference: 0.220, 95% CI: 0.008 to 0.432; P = 0.042, paired t-test). Sensitivity analysis using the Wilcoxon signed-rank test confirmed that BCVA improvement from baseline to 12 months was significantly greater in the study group (Z = −2.068, P = 0.039), consistent with the paired t-test results. Sensitivity analysis using an unstructured covariance structure to account for unequally spaced follow-up visits yielded results consistent with the primary analysis (group-by-time interaction: F = 8.955, P < 0.001).

### 3.5 Postoperative NVI status

The proportion of patients who were NVI-free at 1 week, 1 month, and 12 months was 29/31 (93.5%), 30/31 (96.8%), and 30/31 (96.8%) in the study group, and 15/31 (48.4%), 18/31 (58.1%), and 20/31 (64.5%) in the control group. In the paired analysis, the discordant pair counts (B/C) were 15/1 at 1 week, 13/1 at 1 month, and 11/1 at 12 months (P = 0.001, 0.002, and 0.006, respectively; McNemar’s test, **Table 4**). Among patients with NVI at baseline (18 in the study group and 14 in the control group), regression rates were 94.4% vs. 50.0% at 1 week, 100% vs. 71.4% at 1 month, and 100% vs. 71.4% at 12 months (**Table 4**). Regarding recurrence, among patients with baseline NVI who achieved regression during follow-up, recurrence was observed in 1/18 (5.6%) in the study group and 0/10 (0%) in the control group.

**Table 4:** Postoperative NVI status and regression [n(%)].

Outcome measure	Study group	Control group	Discordant pairs (B/C) *	Risk difference (RD, %)†	*P*-value‡
NVI-free status, n (%)	n=31	n=31			
At 1 Week	29 (93.5%)	15 (48.4%)	15/1	+45.2	0.001
At 1 Month	30 (96.8%)	18 (58.1%)	13/1	+38.7	0.002
At 12 Months	30 (96.8%)	20 (64.5%)	11/1	+32.3	0.006
NVI regression among baseline NVI§	n=18	n=14			
At 1 Week	17 (94.4%)	7 (50.0%)			
At 1 Month	18 (100%)	10 (71.4%)			
At 12 Months	18 (100%)	10 (71.4%)			
NVI Recurrence#					
During Follow-up	1/18 (5.6%)	0/10(0%)	–	–	–

*B = number of pairs where the study group had regression while the control group did not; C = number of pairs where the control group had regression while the study group did not.^†^Risk difference (RD) = (B − C)/31. ^‡^McNemar’s test (exact binomial distribution). ^§^Denominator restricted to patients with NVI at baseline (study: 18; control: 14). Pairwise comparison was not performed due to the small sample size and disruption of matched-pair structure in this subgroup; rates are reported descriptively. ^#^Denominator restricted to patients with baseline NVI who achieved regression during follow-up and were therefore at risk of recurrence. Reported descriptively because the matched-pair structure was disrupted in the at-risk population.

### 3.6 Incidence of complications

After 12 months of follow-up, the total complication rate was lower in the study group (12.9%, 4/31) than in the control group (35.5%, 11/31). In the paired analysis, the number of discordant pairs in which the study group had complications while the control group did not was 4, and the number in which the study group had no complications while the control group did was 11; this difference did not reach statistical significance (P = 0.118, McNemar’s test, **Table 5**). For individual complication types, the study group showed numerically lower rates of hyphema (3.2% vs. 12.9%) and shallow anterior chamber (0.0% vs. 6.5%); however, due to the low event frequencies, formal statistical testing was not performed for individual items (**Table 5**).

**Table 5:** Postoperative complications [n(%)].

Complication	Study group (n=31)	Control group (n=31)	Number of inconsistent pairs (B/C) *	Risk difference (RD, %)	*P*-value†
Hyphema	1 (3.2%)	4 (12.9%)	–	–	–
Transient Hypotony	2 (6.5%)	3 (9.7%)	–	–	–
Shallow Anterior Chamber	0 (0.0%)	2 (6.5%)	–	–	–
Choroidal Detachment	1 (3.2%)	1 (3.2%)	–	–	–
Tube Obstruction	0 (0.0%)	1 (3.2%)	–	–	–
Total Patients with Complications	4 (12.9%)	11 (35.5%)	4/11	−22.6	0.118

*B = number of pairs in which the study group had complications while the control group did not; C = number of pairs in which the study group did not have complications while the control group did. For individual complication types, formal statistical testing was not performed due to the low number of events; only descriptive frequencies are reported (denoted as “—”). ^†^McNemar’s test (exact binomial distribution).

### 3.7 Clinical characteristics of surgical failure cases

During the 12-month follow-up, 9 patients (14.5%) experienced surgical failure: 2 (6.5%) in the study group and 7 (22.6%) in the control group. The characteristics of these 9 failure cases are summarized in **Table 6**. Among the failure cases, the majority were male (77.8%), had poor baseline BCVA > 2.0 LogMAR (66.7%), and had diabetes mellitus (66.7%). Notably, all 2 failure cases in the study group had age ≥ 60 years, BCVA > 2.0 LogMAR, diabetes mellitus, and Stage IV glaucoma, whereas these features were less consistently present in the control group failure cases (42.9%–57.1%). The mean preoperative IOP was higher in the study group failure cases (55.3 mmHg) than in the control group failure cases (46.4 mmHg), though the number of cases was too small for formal statistical comparison.

**Table 6:** Characteristics of the 9 patients with surgical failure.

Characteristic	Study group (n=2)	Control group (n=7)	Total (n=9)
Age (years, mean ± SD)	71.5 ± 9.2	58.6 ± 7.8	61.4 ± 10.1
Age ≥ 60 years, n (%)	2 (100.0)	3 (42.9)	5 (55.6)
Male sex, n (%)	2 (100.0)	5 (71.4)	7 (77.8)
Preoperative IOP (mmHg, mean ± SD)	55.3 ± 1.6	46.4 ± 5.8	48.4 ± 6.5
Preoperative IOP > 55 mmHg, n (%)	1 (50.0)	1 (14.3)	2 (22.2)
Baseline BCVA (LogMAR, mean ± SD)	2.34 ± 0.10	2.08 ± 0.43	2.14 ± 0.38
Baseline BCVA > 2.0 LogMAR, n (%)	2 (100.0)	4 (57.1)	6 (66.7)
Diabetes mellitus, n (%)	2 (100.0)	4 (57.1)	6 (66.7)
Stage IV glaucoma, n (%)	2 (100.0)	3 (42.9)	5 (55.6)
AGV implantation, n (%)	1 (50.0)	3 (42.9)	4 (44.4)

Data are presented as mean ± SD or n (%). AGV, Ahmed glaucoma valve.

## 4 Discussion

As a severe refractory glaucoma, NVG treatment has always been a major challenge in the field of ophthalmology (Selvan et al., 2019). In this PSM-matched retrospective cohort, we evaluated 12-month outcomes of IVR combined with conventional surgery versus surgery alone. The results suggested that the combination regimen was associated with more favorable IOP control and NVI-free status compared with surgery alone, although these were secondary/exploratory endpoints. However, the observed improvement in surgical success rate, while numerically higher, did not reach statistical significance. An exploratory descriptive analysis of the 9 failure cases revealed that most had poor baseline BCVA, diabetes mellitus, and advanced glaucoma stage, though formal prognostic modeling was precluded by the limited number of events. These findings may provide exploratory evidence for the design of future prospective studies on NVG management.

The study group showed consistently lower postoperative IOP and a numerically higher 12-month success rate compared with controls, although the difference in success rate was not statistically significant. Recent systematic reviews and meta-analyses have demonstrated the efficacy and safety of intravitreal ranibizumab as an adjunct to glaucoma surgery in neovascular glaucoma (He et al., 2024; Wang et al., 2024; Santina et al., 2025). The pathological basis of NVG is that retinal ischemia leads to increased VEGF levels, which in turn induces NVI in the anterior segment. These NVI will not only block the outflow channel of aqueous humor, but also be accompanied by fibrous tissue proliferation, leading to Angle closure and increased IOP (Verma et al., 2025). The higher NVI regression rate among patients with baseline NVI in the study group may be related to the anti-VEGF effect of ranibizumab. The observed recurrence rates among patients with baseline NVI who achieved regression were 5.6% in the study group and 0% in the control group. Several factors may have contributed to this difference, including the anti-VEGF effect of preoperative ranibizumab and the possibility that prior PRP partially reduced the ischemic stimulus. However, the small number of recurrence events limits interpretation. Beyond its anti-angiogenic effects, ranibizumab may reduce the risk of intraoperative and postoperative bleeding by inhibiting NVI, creating more favorable conditions for surgical operation (Elwehidy et al., 2019). VEGF is not only a potent angiogenic factor, but also promotes inflammatory cell infiltration and fibrosis process (Ahmad and Nawaz, 2022). In this study, the overall complication rate was numerically lower in the study group (12.9% vs. 35.5%), although the difference did not reach statistical significance (P = 0.118). This trend may be associated with reduced inflammation and fibrosis, which could help maintain filtration tract patency, although this remains speculative. In terms of visual function, BCVA improvement was greater in the study group than in the control group, although this finding should be interpreted with caution given the borderline P value (P = 0.042) and potential confounding factors such as concurrent diabetic macular edema, combined vitrectomy, and the conversion of non-numeric visual acuities to LogMAR values. This improvement may be related to better IOP control, which could reduce optic nerve damage, and possibly to direct anti-VEGF effects on retinal edema. However, these associations require further investigation. However, concurrent diabetic macular edema or combined vitrectomy may have contributed to visual recovery independently of NVG control, and the small sample size precluded subgroup analysis to isolate these effects. In an exploratory descriptive analysis of the 9 failure cases, most patients had poor baseline BCVA (> 2.0 LogMAR), diabetes mellitus, and advanced glaucoma stage, although the small number of events precluded formal prognostic modeling. These observations are hypothesis-generating and warrant confirmation in larger cohorts.

These findings are broadly consistent with previous reports on anti-VEGF therapy in NVG. A systematic review by He et al. (2024) reported that anti-VEGF combined with surgery was associated with sustained IOP reduction, lowered aqueous VEGF levels, and a reduced risk of postoperative hyphema compared with surgery alone. The NVI regression rates among patients with baseline NVI observed in our study are in line with the central role of VEGF signaling in NVG pathogenesis, as elaborated by Ferrara et al (Ferrara, 2016), and with clinical observations by Sydorchuk et al (Sydorchuk and Novytskyy, 2024), who reported neovascularization reduction within 3–5 days after ranibizumab injection and a decrease in IOP from 30.2 ± 5.8 mmHg to 17.3 ± 3.8 mmHg at 7 days post-injection. Li et al. (2022) also demonstrated rapid regression of neovascularization in NVG patients with vitreous hemorrhage following preoperative ranibizumab combined with trabeculectomy and vitrectomy. Regarding complications, our observation of a numerically lower event rate in the study group aligns with the meta-analysis by Wang et al. (2024), which suggested that anti-VEGF therapy may reduce the risk of certain bleeding-related events. However, given the limited sample size, the exploratory nature of these secondary outcomes, and the fact that the difference in complications did not reach statistical significance, these findings should be interpreted with caution.

The findings of this study offer hypothesis-generating observations that may provide exploratory evidence for future research. Although the observed improvements in IOP control and NVI-free status suggest potential benefits, these were secondary endpoints, and the primary endpoint did not reach statistical significance. Therefore, perioperative ranibizumab should be considered exploratory in the context of NVG management and requires confirmation in larger prospective studies. First, the traditional surgical treatment of NVG often fails due to postoperative NVI and fibrosis. Adjunctive ranibizumab may be associated with NVI regression and reduced inflammation, which could influence surgical outcomes; however, the improvement in success rate did not reach statistical significance in this cohort. Second, the observed BCVA improvement in the study group (0.38 ± 0.53 vs. 0.16 ± 0.58 LogMAR; P = 0.042) suggests that ranibizumab may be associated with visual improvement beyond IOP control. This observation suggests that visual function may be an important outcome to assess in future NVG studies, although further research is needed to confirm the impact on quality of life. Third, the descriptive analysis of the 9 failure cases revealed that most had poor baseline BCVA, diabetes mellitus, and advanced glaucoma stage, suggesting that patients with multiple baseline risk factors may benefit from closer postoperative monitoring, although this remains to be confirmed. However, the small number of events precludes definitive conclusions, and these observations are hypothesis-generating only.

This study has several limitations. First, the retrospective, single-center design and relatively small sample size (31 patients per group after PSM) may limit generalizability and statistical power, particularly for low-event-rate outcomes. The 12-month follow-up, while adequate for short-term assessment, is insufficient to evaluate long-term outcomes, and the small sample precluded meaningful subgroup analysis by surgical procedure. Second, although PSM was used to minimize confounding, residual confounding from unmeasured factors cannot be excluded. Third, other core outcome measures for NVG (e.g., visual field progression, quality of life) were not assessed due to the retrospective design. Fourth, protocol-driven postoperative PRP or repeat anti-VEGF injections were not mandated, and data on systemic control of the underlying retinal diseases (e.g., glycemic control) during follow-up were not systematically recorded, both of which may have influenced outcomes. Fifth, although angle status was included in the propensity-score model, residual imbalance persisted (SMD = 0.258), and while we adjusted for this variable in the longitudinal outcome models, standardized gonioscopic assessments for NVA regression or angle structural changes during follow-up were not consistently recorded, precluding analysis of their impact on IOP outcomes. Sixth, the conversion of non-numeric visual acuities to LogMAR values may have introduced measurement variability; however, sensitivity analyses using the Wilcoxon signed-rank test yielded results consistent with the primary analysis, supporting the robustness of the BCVA findings. Seventh, the observed BCVA improvement should be interpreted with caution, as concurrent diabetic macular edema (DME) or combined vitrectomy may have contributed to visual recovery independently of NVG control. Therefore, our findings should be interpreted as exploratory, and larger prospective multicenter studies with longer follow-up and standardized protocols are needed to confirm these results.

## 5 Conclusion

In conclusion, this retrospective matched cohort study observed that IVR combined with conventional surgery was associated with more favorable IOP control and NVI-free status, whereas the 12-month surgical success rate showed a numerical improvement that did not reach statistical significance. These findings are exploratory and hypothesis-generating; larger prospective studies with standardized protocols and longer follow-up are needed to confirm these results.

## Data Availability

The original contributions presented in the study are included in the article/**Supplementary Material**. Further inquiries can be directed to the corresponding author.
