# Supplementary material for: Intravitreal ranibizumab as an adjunct to conventional surgery for neovascular glaucoma: a retrospective observational study of efficacy and clinical characteristics of surgical failure cases
Source: Front Physiol. 2026 Sep 15;17:1915879. doi: 10.3389/fphys.2026.1915879 (PMC13619498; doi:10.3389/fphys.2026.1915879)
Supplement: Supplementary file 1 [file Table1.docx]

**SPSS MIXED Syntax for IOP Analysis:**

MIXED IOP BY Group Time WITH PreopIOP GlaucomaStage AngleNV Age

/CRITERIA=CIN(95) MXITER(100) MXSTEP(10) SCORING(1) SINGULAR(0.000000000001) HCONVERGE(0, ABSOLUTE) LCONVERGE(0, ABSOLUTE) PCONVERGE(0.000001, ABSOLUTE)

/FIXED=Group Time PreopIOP GlaucomaStage AngleNV Age Group*Time | SSTYPE(3)

/METHOD=REML

/PRINT=SOLUTION TESTCOV

/RANDOM=INTERCEPT | SUBJECT(PairID) COVTYPE(VC)

/REPEATED=Time | SUBJECT(PairID*Subj) COVTYPE(AR1)

/EMMEANS=TABLES(Group*Time) COMPARE(Group) ADJ(LSD).

**SPSS MIXED Syntax for BCVA Analysis:**

MIXED BCVA BY Group Time WITH PreopBCVA GlaucomaStage AngleNV Age

/CRITERIA=CIN(95) MXITER(100) MXSTEP(10) SCORING(1) SINGULAR(0.000000000001) HCONVERGE(0, ABSOLUTE) LCONVERGE(0, ABSOLUTE) PCONVERGE(0.000001, ABSOLUTE)

/FIXED=Group Time PreopBCVA GlaucomaStage AngleNV Age Group*Time | SSTYPE(3)

/METHOD=REML

/PRINT=SOLUTION TESTCOV

/RANDOM=INTERCEPT | SUBJECT(PairID) COVTYPE(VC)

/REPEATED=Time | SUBJECT(PairID*Subj) COVTYPE(AR1)

/EMMEANS=TABLES(Group*Time) COMPARE(Group) ADJ(LSD).

**Supplementary Table S1. IOP Mixed Model – Fixed Effects and Convergence**

| Source | Numerator df | Denominator df | F | P-value |
| --- | --- | --- | --- | --- |
| Group | 1 | 56.54 | 17.732 | <0.001 |
| Time | 4 | 239.063 | 71.708 | <0.001 |
| Group × Time | 4 | 239.063 | 16.864 | <0.001 |
| PreopIOP | 1 | 55.558 | 0.234 | 0.631 |
| GlaucomaStage | 1 | 55.558 | 1.028 | 0.315 |
| AngleNV | 1 | 55.558 | 0.405 | 0.527 |
| Age | 1 | 55.558 | 0.139 | 0.711 |

Convergence: -2 restricted log-likelihood = 964.063; converged successfully.

**Supplementary Table S2. IOP Mixed Model – Covariance Parameters**

| Parameter | Estimate | Std. Error | Wald Z | P-value | 95% CI |
| --- | --- | --- | --- | --- | --- |
| AR1 diagonal | 13.215 | 2.378 | 5.556 | <0.001 | 9.287 – 18.804 |
| AR1 rho | 0.972 | 0.006 | 173.718 | <0.001 | 0.959 – 0.981 |
| Intercept [Subject = PairID] variance | 0 | — | — | — | — |

**Supplementary Table S3. BCVA Mixed Model – Fixed Effects and Convergence**

| Source | Numerator df | Denominator df | F | P-value |
| --- | --- | --- | --- | --- |
| Group | 1 | 28.483 | 5.725 | 0.024 |
| Time | 4 | 237.828 | 21.771 | <0.001 |
| Group × Time | 4 | 237.828 | 5.492 | <0.001 |
| PreopBCVA | 1 | 49.239 | 3.054 | 0.087 |
| GlaucomaStage | 1 | 40.587 | 1.463 | 0.233 |
| AngleNV | 1 | 41.715 | 0.951 | 0.335 |
| Age | 1 | 43.468 | 0.108 | 0.745 |

Convergence: -2 restricted log-likelihood = -418.657; converged successfully.

**Supplementary Table S4. BCVA Mixed Model – Covariance Parameters**

| Parameter | Estimate | Std. Error | Wald Z | P-value | 95% CI |
| --- | --- | --- | --- | --- | --- |
| AR1 diagonal | 0.081 | 0.02 | 3.944 | <0.001 | 0.049 – 0.133 |
| AR1 rho | 0.958 | 0.011 | 84.553 | <0.001 | 0.929 – 0.975 |
| Intercept [Subject = PairID] variance | 0.086 | 0.034 | 2.496 | 0.013 | 0.039 – 0.188 |

**Recalculation of Standardized Mean Differences (SMDs) Formulas used:**

1. Continuous variables: Cohen‘s d = (mean_1_ − mean_2_) / √[(SD_1_^2^ + SD_2_^2^)/2]
2. Binary variables: SMD = (p_1_ − p_2_) / √[p̄(1−p̄)], where p̄ = (p_1_+p_2_)/2
